# 2274. Trends in Empiric Antibiotic Utilization for Patients Hospitalized with Community-Onset SARS-CoV-2 Infections and Viral Sepsis During the COVID-19 Pandemic

**DOI:** 10.1093/ofid/ofad500.1896

**Published:** 2023-11-27

**Authors:** Claire N Shappell, Michael Klompas, Sanjat Kanjilal, Christina Chan, Caroline S McKenna, Chanu Rhee

**Affiliations:** Brigham and Women's Hospital, Boston, Massachusetts; Harvard Medical School and Harvard Pilgrim Health Care Institute, Boston, Massachusetts; Department of Population Medicine, Harvard Medical School and Harvard Pilgrim Healthcare Institute, Cambridge, MA; Harvard Pilgrim Health Care Institute, Boston, Massachusetts; Harvard Pilgrim Health Care Institute, Boston, Massachusetts; Brigham and Women's Hospital, Boston, Massachusetts

## Abstract

**Background:**

Empiric antibiotic use in patients hospitalized with COVID-19 was common early in the pandemic despite low rates of bacterial co-infection. Little is known, however, about trends in antibiotic prescribing for patients with COVID-19 with and without viral sepsis.

**Methods:**

We retrospectively identified all adults admitted to 5 Massachusetts hospitals from March 2020 to November 2022 with community-onset COVID, defined by positive SARS-CoV-2 PCR prior to hospital day (HD) 3. Community-onset COVID sepsis was defined as a positive PCR and ≥1 acute organ dysfunction (advanced oxygen support, vasopressors, elevated lactate, or changes in baseline creatinine, bilirubin, or platelets) before HD 3. We calculated quarterly rates of initial (before HD 3) and prolonged (≥4 days/first week) empiric broad-spectrum antibiotics in COVID patients with and without sepsis and estimated quarterly odds for receipt of prolonged antibiotics relative to the first pandemic quarter using logistic regression to adjust for age, comorbidities, and positive blood or sputum cultures before HD 3.

**Results:**

Of 431,017 total hospitalizations, 21,563 (5.0%) had community-onset COVID, of whom 3,829 (17.8%) had COVID sepsis. COVID patients with sepsis had a positive blood or sputum culture before HD 3 in 8.8% of encounters versus 1.8% of those without sepsis. Initial antibiotics were given to 69.0% of COVID patients with sepsis vs 34.3% of those without sepsis and were continued for ≥4 days in 49.5% and 18.6% respectively. Crude quarterly rates of prolonged antibiotic use decreased from 66.9% to 47.6% for COVID patients with sepsis (IRR 0.96/quarter, 95% CI 0.95-0.97) and from 31.0% to 16.4% for COVID without sepsis (IRR 0.95/quarter, 95% CI 0.94-0.96) (**Figure**). Trends in prolonged antibiotic use were similar after risk adjustment (quarterly aOR 0.92 for COVID with sepsis, 95% CI 0.90-0.94 and aOR 0.91 for COVID without sepsis, 95%CI 0.90-0.92).

Trends in Prolonged Antibiotics Prescribing for Hospitalized Patients with SARS-CoV-2 with and without Viral Sepsis

**Figure:**
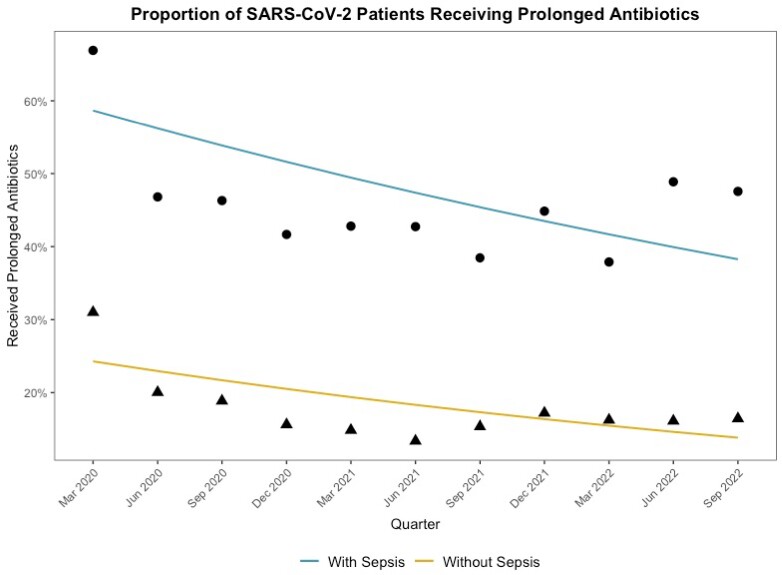

**Conclusion:**

During the first 2.5 years of the pandemic, empiric antibiotics were given to 2/3 of COVID patients with community-onset sepsis and 1/3 of COVID patients without sepsis, usually for at least 4 days, despite low rates of documented bacterial co-infection. Antibiotic use in both groups, however, significantly declined over time.

**Disclosures:**

**Michael Klompas, MD, MPH**, UpToDate, Inc.: Royalties for chapters on pneumonia **Sanjat Kanjilal, MD, MPH**, GSK: Advisor/Consultant|Pattern biosciences: Advisor/Consultant|Roche: Honoraria|Uptodate: Royalties **Chanu Rhee, MD, MPH**, Cytovale: Advisor/Consultant|Pfizer: Advisor/Consultant|UpToDate, Inc.: Honoraria

